# Photothermal‐Activated Antibacterial Amyloid‐Polyphenol‐Iron Hydrogels for Synergistic Wound Healing

**DOI:** 10.1002/adhm.202505910

**Published:** 2026-05-10

**Authors:** Di Wu, Jiangtao Zhou, Yang Shen, Qiyao Sun, Tong Li, Xiaoyang Zou, Bin Liu, Hongshan Liang, Raffaele Mezzenga

**Affiliations:** ^1^ College of Food Science and Technology Huazhong Agricultural University Wuhan Hubei China; ^2^ College of Food Science and Engineering Qingdao Agricultural University Qingdao China; ^3^ Department of Health Sciences and Technology ETH Zurich Zurich Switzerland; ^4^ Department of Food Science & Technology National University of Singapore Singapore Singapore; ^5^ National University of Singapore (Suzhou) Research Institute Suzhou Jiangsu China; ^6^ Department of Materials ETH Zurich Zurich Switzerland

**Keywords:** amyloid fibrils, antibacterial hydrogel, photothermal therapy, polyphenol, wound healing

## Abstract

The design and development of stimuli‐responsive hydrogels with anti‐inflammatory and antibacterial properties have triggered significant progress in wound healing treatment. Herein, we report a thermally triggered supramolecular hydrogel combining potent antibacterial activity with photothermal functionality. The incorporation of Fe^3+^ ions into tannic acid (TA), both bound to lysozyme amyloid fibril (Lys AF) networks, serves a dual role: enabling rapid gelation and conferring efficient photothermal transduction. This dynamic self‐assembly is primarily driven by thermally enhanced metal‐ligand coordination between Fe^3+^ and the amyloid‐polyphenol complex (LT), while hydrogen bonding contributes to network stabilization. This mechanism is amplified at elevated temperatures and under optimized concentrations. Notably, the formed amyloid‐polyphenol‐iron (LTFe) hydrogel exhibits exceptional photothermal conversion efficiency (88.56%), robust cycling stability, excellent biocompatibility, and potent antibacterial efficacy against both Gram‐negative (*E. coli*) and Gram‐positive (*S. aureus*) bacteria. In vivo studies confirmed that LTFe eradicated pathogens, suppressed inflammatory cytokines, and accelerated wound regeneration. Overall, our work introduces a versatile strategy for designing high‐efficiency and high‐biocompatibility NIR‐responsive hydrogel systems.

## Graphical abstract

We report a thermally triggered supramolecular hydrogel (LTFe) formed by Fe^3^^+^ and tannic acid on lysozyme amyloid fibrils. Fe^3^^+^ enables rapid gelation and photothermal transduction with an efficiency of 88.56%. The LTFe hydrogel exhibits excellent biocompatibility, potent antibacterial activity against *E. coli* and *S. aureus*, and in vivo suppresses inflammation and accelerates wound healing.

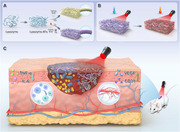

## 1 Introduction

Wound healing is regarded as a complex pathophysiological process involving the phases of hemostasis, inflammatory, cellular proliferation, and matrix remodeling [1, 2]. Current clinical challenges in wound management impose significant health and economic burdens globally [3]. To address this, diverse antibacterial biomaterials have been designed for wound care, including porous cryogels [4], biocompatible membranes [5], and functional hydrogels [6]. Hydrogels, in particular proteinaceous hydrogels, are ideal wound dressings due to their structural mimicry of the extracellular matrix (ECM), biocompatibility, and capacity to maintain a hydrated regenerative microenvironment [7, 8].

Lysozyme amyloid fibrils (Lys AFs), characterized by β‐sheet‐rich architectures, have emerged as promising hydrogel building blocks due to their inherent antimicrobial properties and bioactivity [9]. These highly organized architectures confer exceptional resistance to harsh environments and significantly enhance mechanical stiffness [10, 11]. Notably, Lys AFs exhibit diverse bioactivities arising from their physicochemical similarity to the natural ECM [12], and our prior work reported their excellent performance as bioactive scaffolds for accelerated cell growth and proliferation [13], demonstrating their potential in wound healing. However, hydrogels constructed solely from Lys AFs suffer from suboptimal mechanical strength and limited antibacterial potency, necessitating co‐assembly strategies to enhance their functionality [14, 15, 16].

Naturally occurring bioactive compounds have attracted widespread attention, primarily due to their assembly properties for fabricating advanced functional materials [17, 18]. Among them, tannic acid (TA), a naturally derived polyphenolic compound, offers versatile supramolecular interactions (e.g., hydrogen bonding, hydrophobic effects) via catechol/pyrogallol groups, and facilitates robust protein‐polymer network formation [19, 20, 21, 22]. Remarkably, TA chelates multivalent metal ions (e.g., Fe^3+^) to generate metal‐phenolic networks (MPNs), which enhance mechanical properties [23, 24, 25] and achieve photothermal conversion efficiency (∼40%) surpassing conventional agents [26, 27, 28].

To leverage the inherent bioactivity of Lys AFs as bioactive scaffolds while addressing the limitations of pure Lys AF hydrogels, we developed a synergistic strategy by incorporating TA/Fe^3+^ modules. These components not only served as efficient photothermal agents, but also as dynamic cross‐linkers, facilitating hydrogel formation. Our strategy of systematic design and fabrication of supramolecular hydrogels (denoted LTFe) demonstrated effectiveness as a multifunctional therapeutic platform for eradicating infections, modulating inflammatory responses, and accelerating wound healing.

## 2 Experimental Section

### 2.1 Materials

Hen egg white lysozyme (Lys, L‐6876), tannic acid (TA, 403040), ferric chloride (FeCl_3_, 157740), and Bis‐Tris (B9754) were obtained from Sigma–Aldrich. L929 mouse fibroblast was a generous gift from Prof. Dr. Jean‐Christophe Leroux's laboratory at ETH Zurich.

### 2.2 Preparation of Lys AFs

Lys powder (5 g) was dissolved in Milli‐Q water (45 mL) and stirred for 10 min to acquire a Lys stock solution (10 wt.%). The stock solution was then dialyzed (6–8 kD MWCO, Spectrum Laboratories) against Milli‐Q water at 4°C for another 3 d, followed by lyophilization to obtain purified Lys powder. Lys AFs were fabricated by incubating purified Lys solution (2 wt.%) at 90°C and pH 2 conditions for 8 h with magnetic agitation (350 rpm). The fibrillization was quenched by an ice bath.

### 2.3 Preparation of LTFe Hydrogels

TA and FeCl_3_•6H_2_O were dissolved in DMSO and Milli‐Q water, respectively, to prepare stock solutions (112 mm). Briefly, TA and FeCl_3_•6H_2_O were mixed with 1 wt.% of Lys AFs solution under vortex, followed by incubation under different heating temperatures for gelation. The formulations for fabricating LTFe composites are shown in Table S1. For TFe composites preparation, the method is the same, but Lys AFs were replaced by an equal volume of Bis‐Tris buffer.

### 2.4 Morphology Characterization of LTFe Hydrogels

Transmission electron microscopy (TEM) characterization was carried out using a TFS Morgagni 268 (Thermo Fisher Scientific, USA) with an accelerating voltage of 100 kV. Atomic force microscopy (AFM) characterization was performed by a Bruker Multimode 8 scanning probe microscope (Bruker, USA). AFM imaging was operated in soft tapping mode under ambient conditions, with a commercial silicon nitride cantilever (Bruker, USA) at a vibration frequency of 150 kHz. A relatively soft tip‐sample interaction was applied during the imaging. AFM images were simply flattened using Nanoscope 8.1 software (Bruker, USA). HAADF‐STEM images were captured by a Titan Themis G2 microscope (FEI, USA), equipped with a probe spherical aberration corrector and operated at 300 keV.

### 2.5 Characterization of LTFe Hydrogels

Rheological measurement was performed on a Physica MCR 502 rheometer (Anton Paar, Austria) with a Couette CC17 geometry. Time sweeps were carried out from 0 to 3600 s under different temperatures. Frequency sweeps were examined under different temperatures from 0.1 to 10 rad/s in the linear viscoelastic regime under a fixed strain amplitude of γ = 1%. X‐ray photoelectron spectroscopy (XPS) measurements were performed with an AXIS Ultra^DLD^ apparatus (Kratos, UK). Fourier transform infrared spectroscopy (FTIR) was conducted by a 640‐IR spectrometer (Varian, USA). All samples were scanned over 4000 to 500 cm^−1^ at room temperature with a resolution of 2 cm^−1^. The 2,2‐Diphenyl‐1‐picrylhydrazyl (DPPH) scavenging activity was tested by Ultraviolet–visible absorption spectrum (UV–vis, Agilent Technologies, USA) and calculated according to a previous protocol [29]. Isothermal titration calorimetry (ITC) determination was obtained using a MicroCal PEAQ‐ITC instrument (Malvern, UK). Experiments were performed with TA titration of Lys AFs and Fe^3+^ titration of LT composites. Each injection lasted 20 s with an interval of 150 s under a stirring speed of 500 rpm. UV–vis was tested by a Varian Cary 100 Bio UV–vis spectrophotometer (Agilent Technologies, USA), ranging from 900 to 400 nm.

### 2.6 Molecular Docking and Molecular Dynamics Simulation

Hen egg white lysozyme structure was downloaded from the RCSB Protein Data Bank (1HEW). Molecular docking was performed by AutoDock Vina 1.2.5. Multiple scoring functions were used to calculate and analyze the binding affinity. Molecular dynamics simulations were conducted by the Gromacs 2024.2 package. The opls 2005 force field was employed to parameterize the protein and small molecules, while the TIP3P model was used for the water solvent. The protein‐TFe complex was placed in a cubic water box and solvated. The charge of the system was neutralized by adding 0.150 M chloride and sodium ions. The energy of the system was initially minimized using the steepest descent minimization method for 50 000 steps. Subsequently, the positions of heavy atoms were restrained for NVT and NPT equilibration for an additional 50 000 steps. The system temperature was maintained at 298 or 368 K, and the system pressure was maintained at 1 bar. After completing the two equilibration stages, an unrestricted simulation was performed for 100 ns. The interactions were analyzed, and dynamic trajectory animations were generated using Maestro 2023.

### 2.7 Thermodynamics Properties Characterization of LTFe Hydrogel

0.5 mL of sample was loaded in an open container and irradiated with an 808 nm laser (Ocean Optics, USA) with the laser spot size of ≈30 mm and an irradiation distance of ≈15 cm under different laser powers. A PI 640i infrared thermal camera (Optris, Germany) linked with Optris PIX Connect software was used for image performance. Photothermal stability was evaluated after five cycles of a consecutive laser on/off process at a power density of 1.5 W cm^−2^.

According to the previously reported method [30], the photothermal conversion efficiency (η) was calculated using the following equation:

(1) $$\eta =\frac{hS\left(T_{max}-T_{sur}\right)-Q_{dis}}{I(1-10^{-A_{\lambda }})}$$

where η is the photothermal conversion efficiency of LTFe hydrogel; *T_max_* is the maximum temperature reached by the sample; *T_sur_* is the ambient temperature; *I* is the laser power; *A_λ_* is the absorbance of LTFe hydrogel at the wavelength of 808 nm; *hS* and *Q_dis_* and can be calculated by the following Equations (2) and (3), respectively:

(2) $$hS=\frac{mC_{p}}{\tau }$$

(3) $$Q_{dis}=hS\times \left(T_{maxH_{2}O}-T_{sur}\right)$$

where *m* and *C_p_* are the mass and heat capacity of water, respectively; $T_{maxH_{2}O}$ is the maximum temperature reached by the water; *T_sur_* is the ambient temperature; *θ* can be calculated by the following Equations (4)–(6):

(4) $$t=\tau ln\;\frac{T-T_{sur}}{T_{max}-T_{sur}}=\tau ln\frac{\Delta T}{\Delta T_{max}}$$

(5) $$\mathrm{If}\;\theta =\frac{\Delta T}{\Delta T_{\max}}$$

(6) $$t=\tau \left(-Ln\theta \right)$$

where T are temperatures at different time points during cooling. *τ* is the slope of the linear time data from the cooling period vs. ‐*Lnθ*.

In conclusion, $\eta =\frac{mC_{p}\left(\frac{\Delta T_{\mathrm{Sample}}}{\tau }-\frac{-\Delta T_{H_{2}O}}{\tau }\right)}{I\left(1-10^{-A}\lambda \right)}$where Δ*T_sample_* and $\Delta T_{H_{2}O}$ are the temperature change of samples being studied and pure H_2_O, respectively.

### 2.8 In Vitro Antibacterial Activity

*E. coli* (Gram‐negative bacteria) and *S. aureus* (Gram‐positive bacteria) were used to estimate the in vitro antibacterial performance. Prior to each experiment, all samples were sterilized by UV light for 2 h. Single bacterial colony was collected and incubated overnight (approximately 16 h) at 37°C in lysogeny broth (LB). The bacteria were washed with sterile PBS and diluted to an appropriate concentration for the following experiments. 50 µL of diluted bacteria suspension (10^4^ CFUs/mL) was mixed with different formulations with/without near‐infrared light (NIR) irradiation (808 nm, 1.5 W cm^−2^, 10 min), and the spread plate method was employed to evaluate the antibacterial effect. For SEM observation, the bacteria suspension (10^8^ CFUs/mL) was incubated with different formulations with/without NIR irradiation for 5 h at 37°C. The bacteria were then washed with PBS and fixed with 2.5% glutaraldehyde overnight at 4°C. Dehydration was conducted, involving a gradient of ethanol aqueous solutions (30, 50, 70, 95, 100 v/v%) for 15 min, before freeze‐drying. Bacterial morphology was further determined by scanning electron microscopy (SEM) (S‐4800, Japan) under a 5 kV accelerating voltage. DCFH‐DA assay was conducted using a Beyo3D ROS Assay Kit (Beyotime, China) following the manufacturer's protocol. The fluorescence intensity was measured using a SPARK multimode microplate reader (Tecan, Switzerland) with excitation at 488 nm and emission at 525 nm.

### 2.9 Cell Culture

L929 mouse fibroblast cells were cultured in Dulbecco's Modified Eagle Medium (DMEM), supplemented with 10% (v/v) of Fetal Bovine Serum (FBS) and 1% (v/v) of penicillin‐streptomycin, and incubated in a humidified incubator (Thermo Scientific, USA) at 37°C under an atmosphere of 5% CO_2._ Cells were allowed to reach 80% confluence and then harvested by trypsinization before subculture. Prior to cell cultivation, all samples were sterilized by UV irradiation for at least 2 h.

### 2.10 Cytotoxicity Assay of LTFe Hydrogel

For MTS assay, 1 × 10^4^ cells were seeded in 96‐well plates and incubated for 24 h. The culture medium was replaced by fresh culture medium containing different formulations (Lys, TFe, LTFe). After an incubation of 24 h, MTS reagent was added and incubated for another 4 h. Cell viability was detected by a microplate reader (Tecan, Austria) and calculated as the percentage of viable cells normalized to unexposed controls. For live/dead assay, LTFe hydrogels were placed on 8‐µ plates (µ‐Slide 8 Well lbiTreat, Ibidi). 5 × 10^4^ L929 cells were seeded onto hydrogels and incubated for 24 h, followed by incubating cells with live/dead reagents (2 µm calcein AM and 4 µm ethidium homodimer‐1, Invitrogen). The images were captured with a Zeiss LSM 780 microscope (Zeiss, Germany). Corresponding quantitative analysis was carried out using the software ImageJ.

### 2.11 In Vivo Wound Healing Evaluation

Ethical approval was obtained from the Laboratory of Animal Experimental Ethical Inspection of the Laboratory Animal Centre, Huazhong Agriculture University, with the assigned approval number: HZAUMO‐2024‐0316.

6‐week female Balb/C mice were randomly assigned to five groups to establish the wound healing model. All mice were raised under SPF conditions and received care according to the guidelines of the Committee on Animal Research and Ethics. Mice were anesthetized with 1% pentobarbital sodium, and full‐thickness wounds (≈1 cm diameter) were created in the back of the mice. Wound infection was established by inoculating 50 µL of *S. aureus* (10^8^ CFUs/mL) on the wound for 1 d. Then, infected wounds were treated with 100 µL of PBS (control), Lys AFs, TFe complex, and LTFe hydrogel with (NLTFe) or without (LTFe) NIR irradiation (808 nm, 1.5 W cm^−2^, 10 min), respectively. The wound areas were photographed at predetermined time points and further calculated by ImageJ software. The antibacterial effect of each group was estimated by the spread plate method at day 1. At day 3 and 11, the skin tissues around infected wounds were dissected from the mice for hematoxylin and eosin (H&E) staining. Immunohistochemistry staining and ELISA assay for TNF‐α and IL‐1β were detected at day 3, while vascular endothelial growth factor (VEGF) and CD31 were tested at day 11.

### 2.12 Statistical Analysis

All data were presented as mean ± SD. Statistical analysis was conducted by IBM SPSS statistics software. One‐way ANOVA was used for multiple comparisons. Statistical differences were defined as: ^*^ *p* < 0.05, ^**^ *p* < 0.01, and ^***^ *p* < 0.001, while ^#^ *p* < 0.05, ^##^ *p* < 0.01, and ^###^ *p* < 0.001 vs. the control group, and the notes of special labels were all given in related experiments.

## 3 Results and Discussion

### 3.1 Characterization of LTFe Hydrogels

**FIGURE 1:**
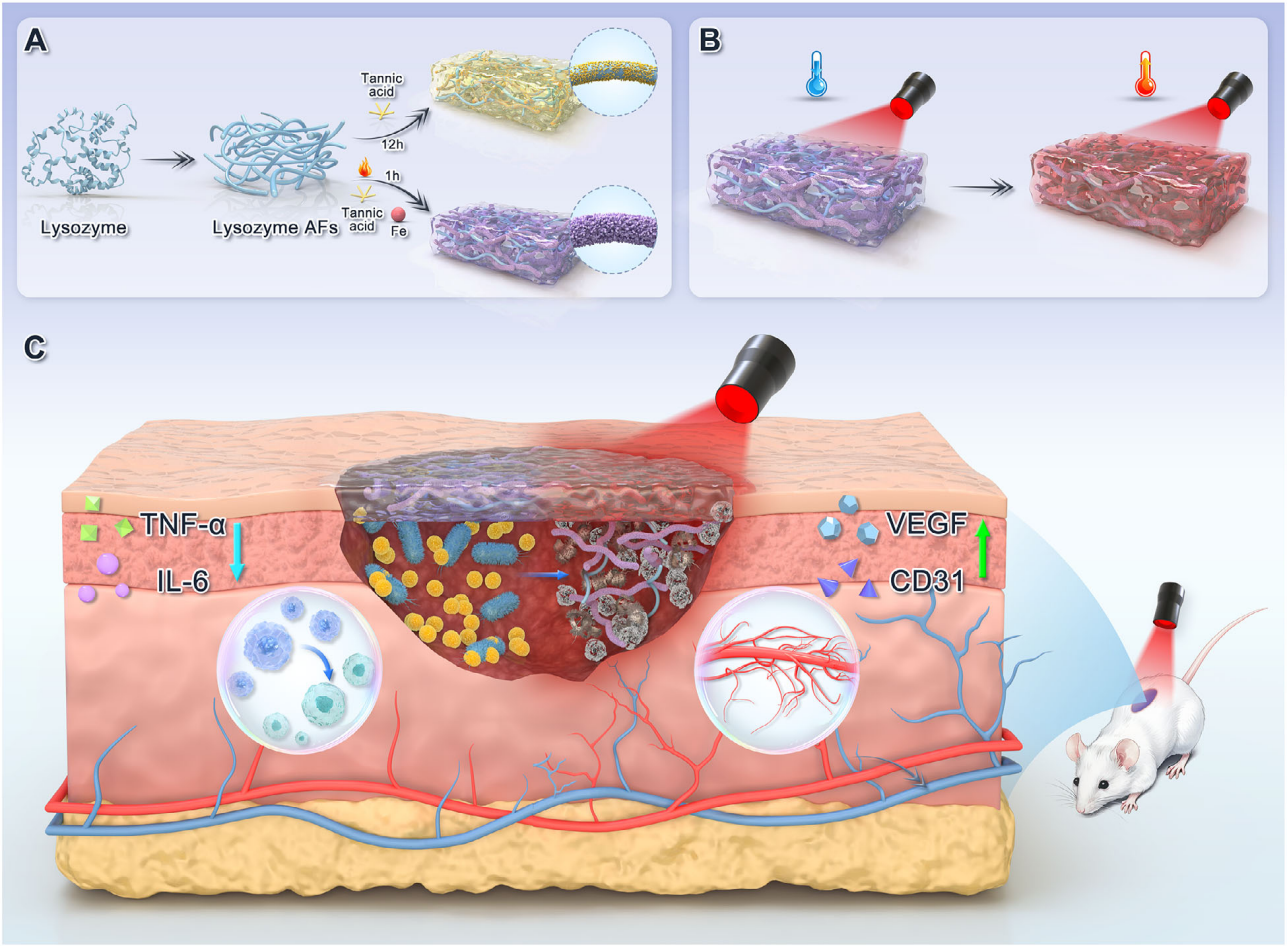
(a) Schematic illustration of the preparation process of LT and LTFe hydrogels, and (b) the photothermal functionality of LTFe hydrogels under NIR laser irradiation. (c) Schematic diagram of the LTFe hydrogel in wound healing application. All schematic diagrams were created using 3ds Max software (Autodesk, Inc., USA).

Our previous investigation revealed that polyphenols with diverse molecular weights were capable of interacting with Lys AFs, forming hydrogels overnight at ambient temperature [31]. These hydrogels served as effective cellular scaffolds with enhanced cell adhesion properties. However, this previous work also identified some critical limitations: hydrogel formation required both a high polyphenol concentration (5.6 mm) and a prolonged gelation time (≥ 12 h). Remarkably, in the present work, we found that the addition of trace amounts of metal ions (Fe^3+^), coupled with controlled heating, dramatically accelerated the gelation kinetics while substantially reducing the required polyphenol concentration (0.175 mM) (Figure 1a). Importantly, the resulting metal‐phenolic coordination not only facilitated rapid network assembly, but also endowed the Lys AFs‐based hydrogel with unique photothermal responsiveness (Figure 1b), demonstrating its efficacy as a multifunctional platform for wound healing treatment (Figure 1c).

**FIGURE 2:**
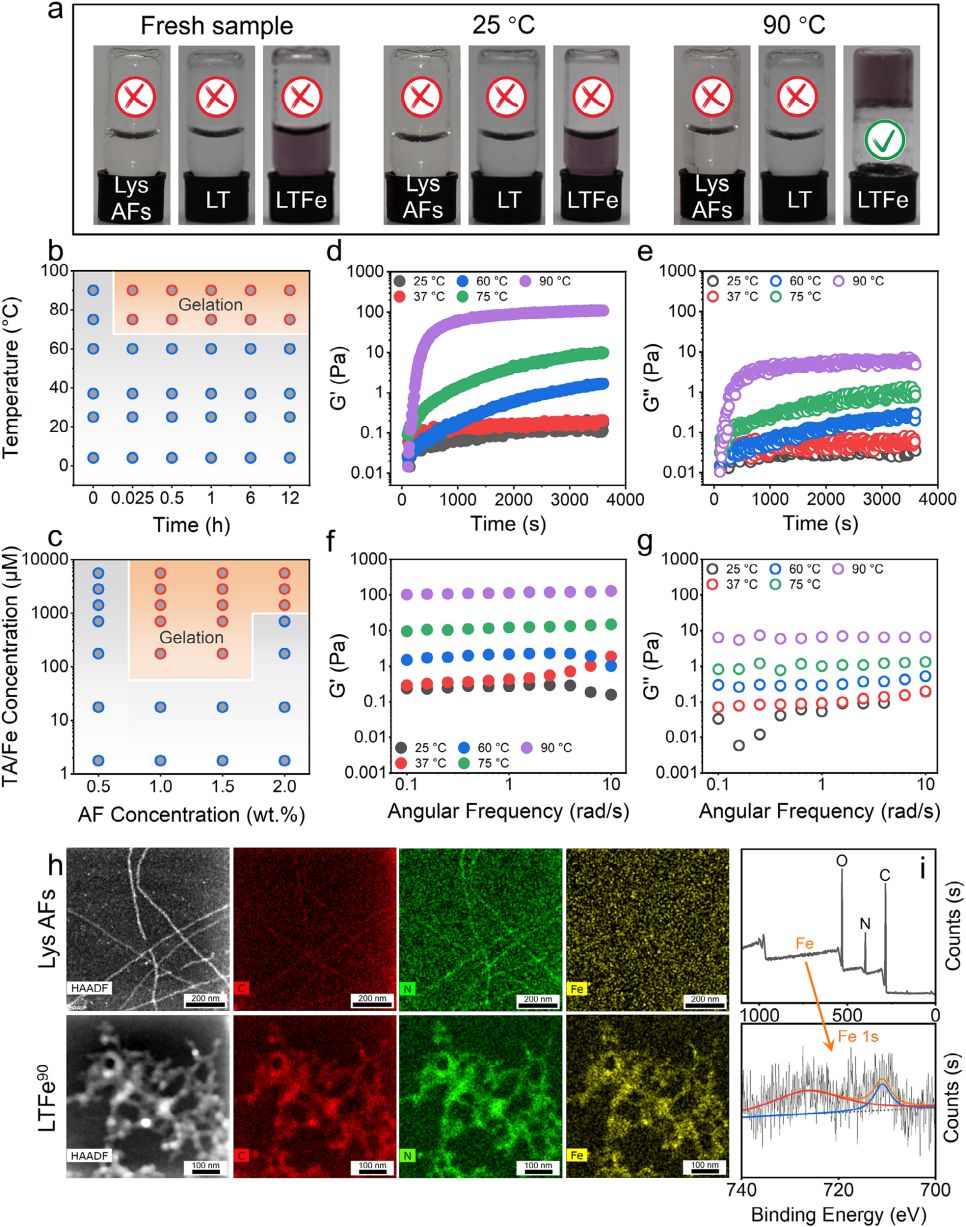
(a) Upturn tests of Lys AFs, LT, and LTFe hydrogels before and after heating at 25°C and 90°C for 1 h. (b) Temperature phase diagram against time for gelation of LTFe hydrogels. (c) Concentration phase diagram for gelation of LTFe hydrogels at 90°C. Storage modulus G′ (solid symbols) and loss modulus G″ (open symbols) as a function of time (d,e) and frequency (f,g) of LTFe_2_ hydrogels under different heating times. (h) HAADF‐STEM and elemental mapping images and (i) XPS spectra of LTFe_2_ hydrogel. Here, LTFe_2_ hydrogels contain 1% of Lys AFs concentration and 0.175 mm of TFe.

Optimal gelation conditions were determined by a visual observation of gelation times (Figure 2a–c). An upturn test showed that the combination of Lys AFs (1 wt.%, ca. 700 µM monomeric concentration) and TA (0.175 mM) failed to form hydrogels (Figure 2a), with the exception of a weak gel behavior under heating conditions, which ranged from 37°C to 90°C for 12 h (Figure S1). The introduction of Fe^3+^ had a negligible effect on gel behavior without heating (Figure 2a); however, the incorporation of Fe^3+^ enabled rapid gelation through metal‐phenolic coordination. Upon heating the precursor solution to 75°C or above, stable LTFe hydrogels could be effectively constructed within 1 h (Figure 2b; Figure S1). This difference in gel behavior presumably arose because of the thermal‐induced dynamic equilibrium of intermolecular interactions, which allowed for the reconstruction of supramolecular structures [32]. It is important to note that the presence of Fe^3+^ was essential for rapid gelation. In contrast, thermal treatment alone without Fe^3+^ resulted in only a weak gel even after prolonged heating (12 h). Stable hydrogels required a Lys AFs concentration equal or above 1% (Figure 2c), regardless of the ratio of TA to Fe^3+^ (Figure S2). Our results illustrated that thermal (90°C)‐triggered hydrogel exhibited rapid crosslinking and gel formation within 1 h (Figure 2b). Additionally, LTFe could still remain in a gelation phase after cooling to room temperature. The gelation process was also detected by rheological analysis. As depicted in Figure 2d,e, the storage moduli (G′) increased and surpassed the loss moduli (G″) within 160 s. It is worth noting that temperature was critical to the gelation rate. Besides, the concentration of TFe composites (Figure S3) and the ratio of TA to Fe^3+^ (Figure S4) also affected the gelation rate and gel strength. Specifically, the temperature increase was accompanied by a rapid gelation process, and almost matched the upturn tests. The equilibrium G′ reached the maximum at 90°C. Frequency sweeps were further performed after hydrogel formation (Figure 2f,g). Results showed that G′ was always higher than the corresponding G″, indicating a solid‐like elasticity. Images captured by high‐angle annular dark‐field scanning transmission electron microscopy (HAADF‐STEM) revealed that 90°C‐triggered LTFe hydrogel exhibited morphological alterations, in comparison to pure Lys AFs (Figure 2h). Additionally, the distribution of Fe in LTFe hydrogel overlapped with that of C and N involved in Lys AFs, as detected by the energy‐dispersive X‐ray spectroscopy (EDX) characterization. To further confirm the formation of LTFe hydrogel, X‐ray photoelectron spectroscopy (XPS) analysis of LTFe hydrogel was performed, as shown in Figure 2i. Fe 1s XPS spectrum displayed two characteristic peaks at 710.9 and 725.9 eV, corresponding to Fe 2p_3/2_ (711 eV) and Fe 2p_1/2_ (725 eV), respectively [33]. Therefore, it appeared that Lys AFs were a bridging template, while high temperature and the TFe module had essential roles for hydrogel formation.

**FIGURE 3:**
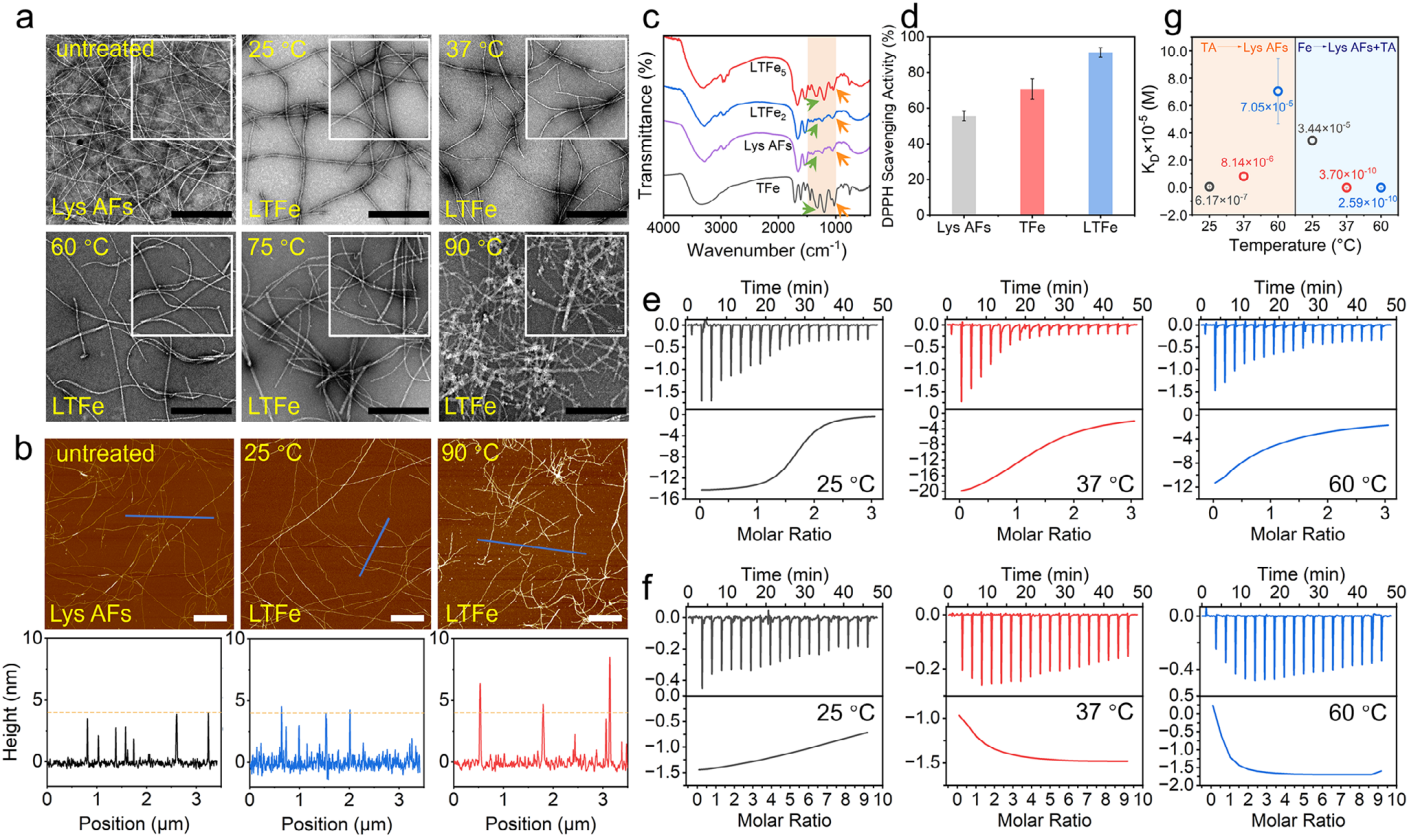
(a) TEM images of Lys AFs and LTFe_2_ hydrogels before and after heating during different temperatures. Bar: 500 nm. (b) AFM images and corresponding height profiles (blue lines) of Lys AFs and LTFe_2_ hydrogels. Bar: 1 µm. (c) FTIR spectra of Lys AFs and LTFe_2_ and LTFe_5_ hydrogels. (d) DPPH scavenging activity of Lys AFs, TFe hybrids, and LTFe_2_ hydrogels. Thermogram (top panels) and binding isotherms (bottom panels) corresponding to the titration of (e) TA (400 µm) with Lys AFs (25 µm) and (f) Fe^3+^ (1200 µm) with Lys AFs (25 µm) and TA (400 µm) hybrids. (g) Comparison of binding affinities (K_D_) of TA to Lys AFs and Fe^3+^ to Lys AFs‐ TA hybrids during different temperatures. Here, LTFe_2_ hydrogels contain 1% of Lys AFs concentration and 0.175 mm of TFe. LTFe_5_ hydrogels contain 1% of Lys AFs concentration and 1.4 mm of TFe.

Pure Lys AFs exhibited relatively uniform fibrillar shapes with an average contour length of several microns, as determined by TEM (Figure 3a) and AFM (Figure 3b). In contrast, the introduction of TFe led to strong metal‐phenolic coordination in LTFe hydrogel, which could be further intensified by high temperature. Briefly, the diameter of LTFe sample at 25°C (approximately 4.5 nm) was slightly higher than pure Lys AFs, as indicated by their random distribution on AF surfaces (Figure 3a,b). However, the fibrils in the LTFe hydrogel after heat treatment (90°C) presented vigorous cross‐linking between AFs (Figure S5) and a significantly increased thickness up to 8.5 nm for LTFe_2_ hydrogel (Figure 3b). Fourier transform infrared spectroscopy (FTIR) suggested that the absorption bands at 1252, 1536, and 1655 cm^−1^ were attributable to the amide I, amide II, and amide III of Lys AFs, respectively [34]. The peaks at 1445, 1320, 1200, 1079, and 1029 cm^−1^ were assigned to the characteristic signatures of TFe composites (Figure 3c) [35]. These results confirmed the successful binding of Lys AFs and metal‐phenolic networks. As shown in Figure 3d, the DPPH scavenging rates of Lys AFs, TFe composites, and LTFe hydrogels were 55.7%, 70.8%, and 91.2%, respectively, demonstrating that the LTFe hydrogel exhibited superior antioxidant capacity.

An in‐depth examination of the thermodynamic properties was performed by ITC measurement. Successive exothermic signals were observed in Figure 3e,f for all formulations. The dissociation constant K_D_ quantifies the stability of a complex, where values below 10^−6^ indicate strong interactions [36]. In our cases, increasing the temperature from 25°C to 60°C resulted in a significant increase in K_D_ values from 6.17 × 10^−7^ to 7.05 × 10^−5^, indicating that elevated temperatures weakened the binding affinity between Lys AFs and TA, with the weakest interaction observed at 60°C (Figure 3g). An opposite change was observed for the titration of Fe^3+^ to LT hybrids (Figure 3g), with K_D_ values of 3.44 × 10^−5^ for LTFe at 25°C, 3.7 × 10^−10^ for LTFe at 37°C^,^ and 2.59 × 10^−10^ for LTFe at 60°C, which corresponds to strong interactions at high temperature. Taken together, these ITC results revealed a temperature‐dependent change in binding dominance: the hydrogen‐bonding‐mediated interaction between Lys AFs and TA weakened at elevated temperatures, while the coordination between Fe^3+^ and the LT hybrid was strongly enhanced. Therefore, the thermally triggered gelation of LTFe hydrogels is mainly attributed to the intensified metal‐ligand coordination, with hydrogen bonding playing an important role in stabilizing the supramolecular network after cooling.

**FIGURE 4:**
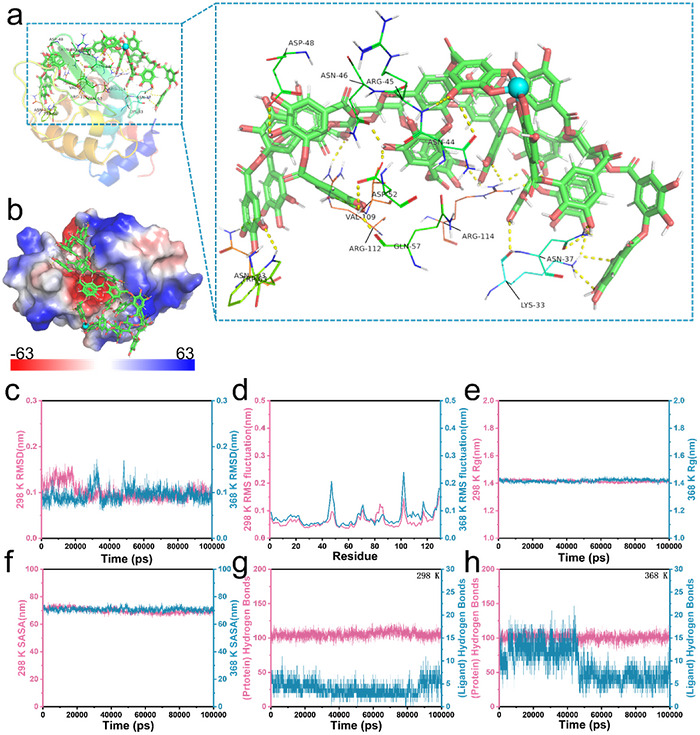
(a) Predicted binding patterns of Lys with TFe. (b) Electrostatic potential analysis diagram. (c) RMSD plot. (d) RMSF plot. (e) Rg plot. (f) SASA plot. (g) The number of hydrogen bonds at 298 K. (h) The number of hydrogen bonds at 368 K.

Molecular docking is an effective strategy to evaluate protein‐ligand interaction patterns and binding affinities. To gain an understanding of the binding energies between the primary sequence of Lys and TFe, we navigate the energy landscape of folded Lys exposed to TFe in solution. As shown in Figure 4a, the TFe ligand formed hydrogen bonds with various Lys residues, such as ARG112, ASP48, ASN46, and ARG45, which indicated that the TFe ligand exhibited high affinity toward Lys. Considering the electrostatic map of the system (Figure 4b), it can be seen that the TFe ligand binds the surface of lysozyme independently of the electrostatic potential, inferring that hydrogen bonds are the dominating supramolecular interaction.

To derive the thermodynamic assembly behavior of the Lys residues with TFe ligand, molecular dynamics simulations were conducted under two different temperatures (298 and 368 K). The system stability was evaluated by the root mean square deviation (RMSD) curve (Figure 4c). Results revealed that the complex structures reached an extremely stable state after 20 ns at 298 K. In contrast, complexes tended toward stability after 70 ns at 368 K. Meanwhile, the root mean square fluctuation (RMSF) graphs of the samples at different temperatures were obtained (Figure 4d). It can be observed that the overall fluctuation of the RMSF of the complex at 368 K was significantly higher than that at 298 K. Based on the above results, it can be concluded that high temperature would disrupt the stability of the system. We further analyzed the radius of gyration (Rg), solvent accessible surface area (SASA), and the number of hydrogen bonds of the system at 298 and 368 K (Figure 4e–h). It was evident that with the temperature increasing, the Rg and SASA of the system slightly increased, while the number of hydrogen bonds decreased significantly. This indicated that the structure of the system was loose at high temperatures, and the intermolecular interactions were disrupted. Moreover, binding free energy calculation (Table S2) illustrated that LTFe composite possessed a preferential binding affinity at 298 K (−38.18 kcal/mol) over 368 K (−27.93 kcal/mol), suggesting superior stability of 298 K.

### 3.2 Photothermal Performance and Photothermal‐Activated Antibacterial Properties of LTFe Hydrogel

**FIGURE 5:**
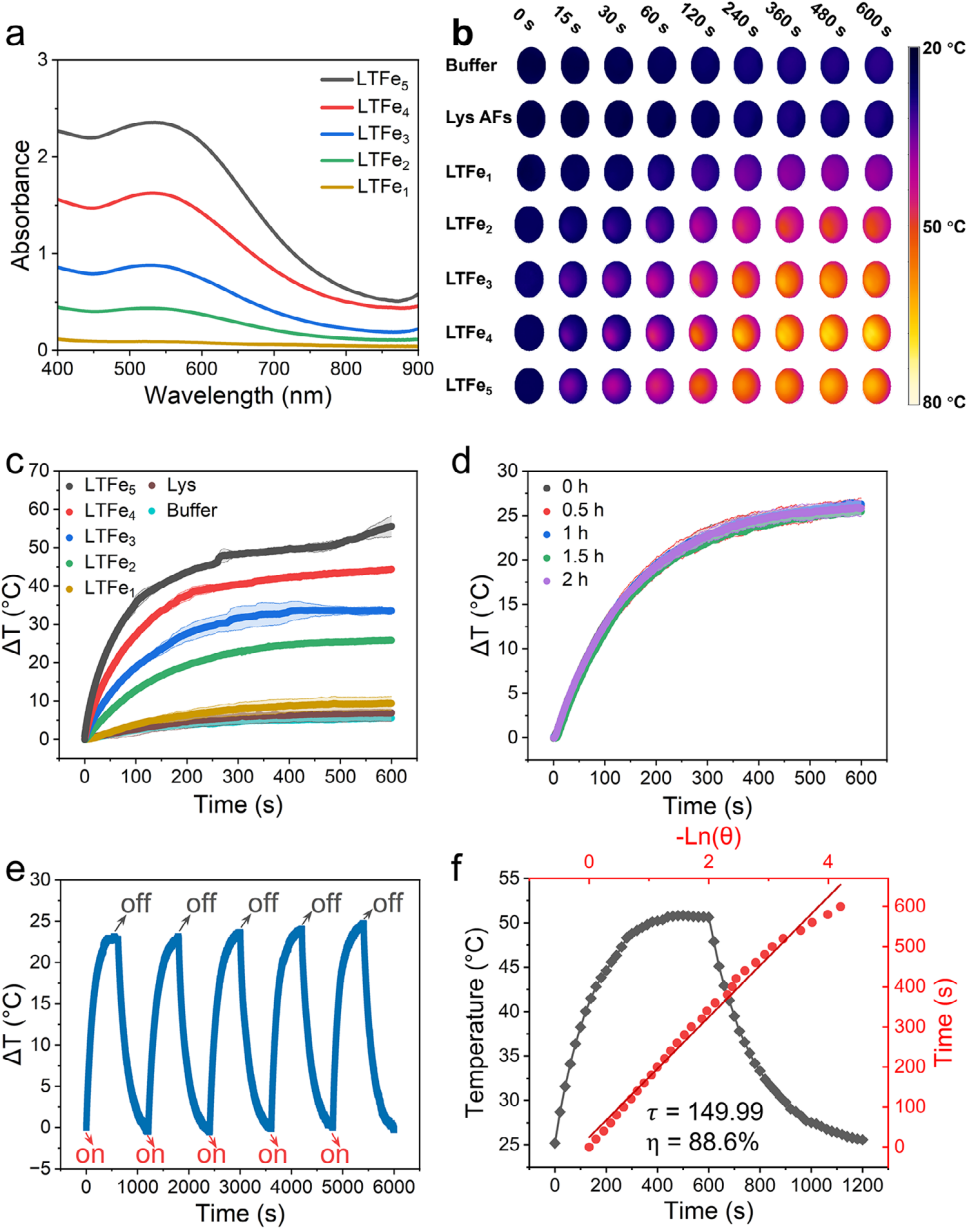
(a) UV–vis absorption spectrum of LTFe hydrogels under different TFe concentrations. (b) Photothermal images and (c) photothermal conversion of Lys AFs and LTFe hydrogels at 1% of Lys AFs and different TFe concentrations from 0.0175 to 1.4 mm under 808 nm laser irradiation (1.5 W cm^−2^). (d) Photothermal conversion of LTFe_2_ hydrogels after UV irradiation for 0, 0.5, 1, 1.5, and 2 h under 808 nm laser irradiation (1.5 W cm^−2^). (e) Photothermal stability study of LTFe_2_ hydrogels for five on/off cycles. (f) Irradiation‐cooling curve of LTFe_2_ hydrogels and linear fit of time/−ln (𝜃) during the cooling process under 808 nm laser irradiation (1.5 W cm^−2^).

Ultraviolet–visible spectroscopy demonstrated a typical characteristic polyphenol‐metal charge transfer peak at ≈550 nm, indicative of the coordination between TA and Fe^3+^ (Figure 5a) [35]. In addition, the absorbance in the visible‐near infrared region displayed an obviously increasing trend with a concomitant increase in TFe concentration from 0.0175 to 1.4 mm (Figure 5a). Infrared images showed that the magnitude of the photothermal response of LTFe hydrogels could be significantly promoted by increasing the relative mass of the TFe module (Figure 5b), which corresponds to the superior photothermal conversion capability. Upon NIR laser irradiation (808 nm, 1.5 W cm^−2^) for 10 min, LTFe hydrogels exhibited rapid temperature increase, depending on TFe concentration from 0.0175 to 1.4 mm (Figure 5c). Specifically, pure Lys AFs and LTFe_1_ hydrogel only fluctuated slightly. In contrast, obvious temperature variations (ΔT) of 25.8°C (LTFe_2_), 33.5°C (LTFe_3_), 44.4°C (LTFe_4_), and 55.5°C (LTFe_5_) were observed for LTFe_2_ to LTFe_5_ hydrogels, respectively, within 10 min NIR irradiation. We thus concluded that a target temperature of 50°C was a suitable temperature for photothermal therapy (PTT) treatment, with the aim to denature bacterial enzymes and proteins for bacterial death, as well as promote angiogenesis and blood vessel density of the granulation tissue for wound healing [37]. Therefore, LTFe_2_ hydrogel, with the maximum temperature at ≈51°C, was applied as the ideal PTT formulation. It is worth noting that LTFe_2_ hydrogel possessed an uncompromised photothermal performance under UV stress (Figure 5d), a remarkable photothermal repeatability of at least five cycles of NIR exposure (Figure 5e), and a good photothermal conversion efficiency of 88.56% (Figure 5f), thus establishing the foundation for PTT‐based treatment for wound healing.

### 3.3 In Vitro Antibacterial Activity of LTFe Hydrogel

**FIGURE 6:**
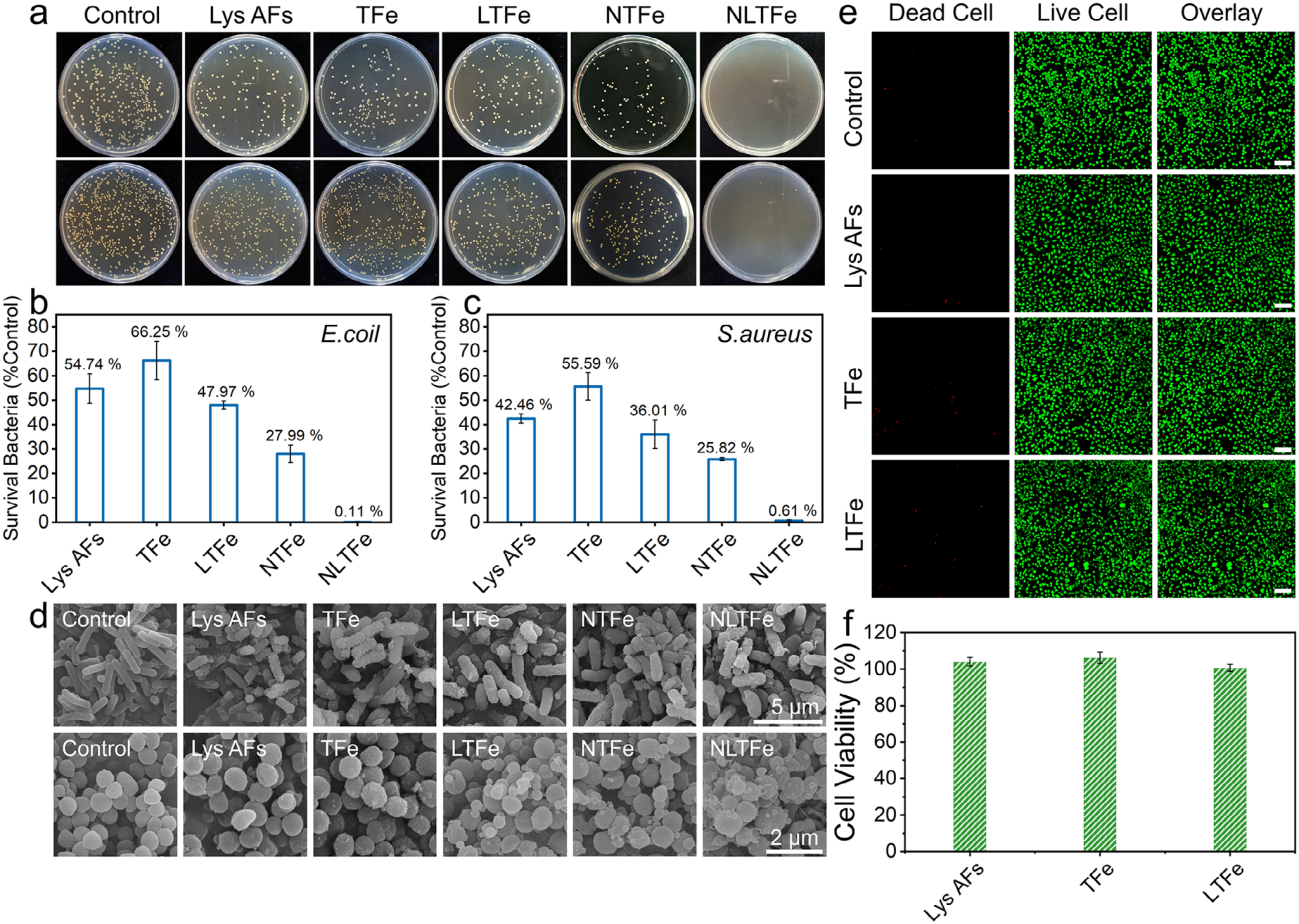
(a) Agar plate pictures and corresponding statistical data of colonies of *E. coli* (b) and *S. aureus* (c) treated with different formulations. (d) SEM image of normal and treated *E. coli* and *S. aureus*. (e) Calcein‐AM/PI stained L929 cells after 24 h incubation with various formulations. (f) Cell viability (MTS assay, *n* = 6) of L929 treated with different formulations. Scale bars: 100 µm.

Preventing bacterial infection is a major challenge in wound healing. Here, two common pathogenic bacteria, *E. coli* (Gram‐negative bacteria) and *S. aureus* (Gram‐positive bacteria), were used to evaluate the antibacterial performance. As depicted in Figure 6a, compared to the control group (bacteria survival rate of 100%), the survival rates of bacteria significantly decreased in Lys AF and TFe groups, due to the antimicrobial effect of abundant lysozyme [38] and catechol groups derived from tannic acid [39]. Nearly 53% (against *E. coli*) and 64% (against *S. aureus*) reductions in bacterial viability were observed for LTFe hydrogel (Figure 6b,c). These results illustrated that both Lys AFs and TFe exhibited effective bactericidal activity toward Gram‐positive microorganisms. Notably, the treatment with LTFe hydrogel in combination with NIR irradiation (NLTFe group) for 10 min led to over 99% killing of both *E. coli* (Figure 6b) and *S. aureu*s (Figure 6c), whereas the TFe complex under the same NIR irradiation (the TFe group) exhibited markedly weaker antibacterial activity. This suggests a superior photothermal antibacterial capacity of the NLTFe hydrogel. Corresponding bacterial morphology observations are further presented in Figure 6d, in which microbes in the control group displayed smooth surfaces and intact structures. However, both groups of bacteria treated with NLTFe became wrinkled, collapsed, and blurred, indicating that NLTFe treatment resulted in bacterial destruction without bacterial species specificity. Of note, intracellular ROS levels were further detected using the DCFH‐DA method in both *E. coli* and *S. aureus* (Figure S6). A marked increase in ROS levels was observed in both bacterial strains (752% in *E. coli* and 619% in *S. aureus* relative to the control), demonstrating that NIR irradiation effectively triggers robust reactive oxygen species (ROS) generation. This was presumably due to the fact that heating process enhanced the Fenton‐like catalytic activity of the TFe complex, which produces highly cytotoxic ROS and contributes to the antibacterial activity. Overall, the outstanding antibacterial performance of the NLTFe hydrogel arises from a synergistic combination of three factors: (i) the inherent antimicrobial activity of Lys AFs and TA, (ii) the direct photothermal bactericidal effect of the TFe complexes under NIR irradiation, and (iii) the heat‐enhanced Fenton‐like catalytic activity of the TFe network, which generates high levels of ROS. This triple mechanism collectively ensures potent and broad‐spectrum bacterial killing, establishing the foundation for long‐term application in the field of antibacterial hydrogel biomaterials.

### 3.4 Biocompatibility of LTFe Hydrogel

The in vitro biocompatibility of different formulations (Lys AFs, TFe, LTFe_2_ hydrogel) was evaluated by cytotoxicity assay of fibroblast cells (L929). It is clearly evident from the live/dead assay results that L929 cells were mostly alive (green) and scattered throughout the hydrogel (Figure 6e). Corresponding quantitative results were obtained by MTS assay, which displayed the same phenomenon (Figure 6f). Briefly, cell viabilities reached almost 100% with different treatments after 24 h of incubation, thus corroborating their good biocompatibility.

### 3.5 *S. aureus*‐Infected Skin Wound Healing Evaluation of the NLTFe‐Based Hydrogel

**FIGURE 7:**
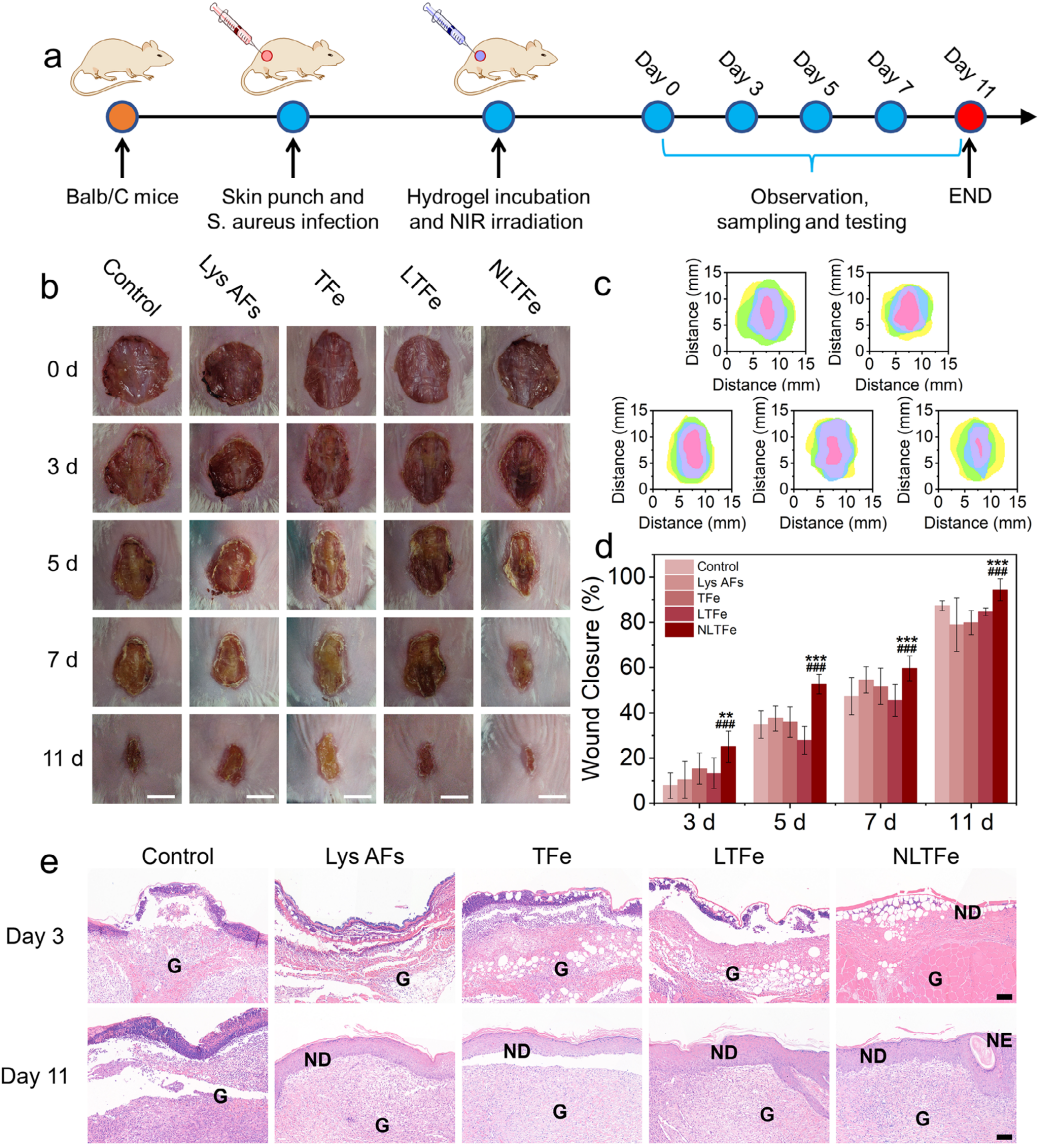
(a) Schematic illustration of *S. aureus*‐infected wound healing mice model. (b) Photographs of wounds and (c) corresponding schematic diagram of wound healing process at 0, 3, 5, 7, and 11 d (from left to right) for control (first row), Lys AFs (first row), TFe composite (second row), LTFe‐ and NLTFe‐based hydrogels (second row). Horizontal/vertical scale bars: 5 mm. (d) Wound closure ratio after *S. aureus* infection at 3, 5, 7, and 11 d. (e) Histological (H&E staining) images of infected wounds in different treatment groups: saline (control), Lys AFs, TFe composite, LTFe‐ and NLTFe‐based hydrogels. Scale bars: 100 µm. *Note*: ^*^ *p* < 0.05, ^**^ *p* < 0.01, and ^***^ *p* < 0.001 vs. LTFe hydrogel; ^#^ *p* < 0.05, ^##^ *p* < 0.01, and ^###^ *p* < 0.001 vs. the control group, *n* ≥ 5.

An animal model of *S. aureus*‐infected wounds was used to explore the antibacterial, anti‐inflammatory, and healing‐acceleration performance of NLTFe‐based hydrogels. A full‐thickness wound was created in the back of Balb/C mice and inoculated with *S. aureus*, followed by treatment with saline control, Lys AFs, TFe composite, LTFe‐, and NLTFe‐based hydrogels. The observation, sampling, and testing operations were executed at 0, 3, 5, 7, and 11 d (Figure 7a). Representative wound images and schematic diagrams of wound closure progression revealed that NIR‐assisted LTFe hydrogel yielded a predominant wound repair advantage compared to other groups (^***^ *p* < 0.01) (Figure 7b,c). Corresponding quantitative results of wound closure ratios were 7.9%, 10.5%, 15.3%, 13.3%, and 25.1% at day 3 postoperative for the control, Lys AFs, TFe composite, LTFe, and NLTFe hydrogels, respectively (Figure 7d), suggesting that NLTFe hydrogel possessed a rapid wound healing capability. Notably, the wounds treated with NLTFe hydrogel had mostly recovered (wound closure rate of 94.4%) at 11 d, in comparison with other groups (Figure 7d). As expected, LTFe_2_ hydrogel with NIR irradiation displayed almost no contamination after 1 d, while serious bacterial infection occurred in other groups (Figure S7), which is consistent with the in vitro antibacterial results. Histomorphological analysis of *S. aureus*‐infected skin tissue was subsequently carried out to investigate the therapeutic efficacy in different treatments (Figure 7e). As shown in H&E staining images, the control group exhibited an active inflammatory response on day 3. In contrast, the inflammatory cell infiltration could be effectively suppressed in the NLTFe group, in comparison to other treatment groups. This was ascribed to the superior synergistic anti‐infective and anti‐inflammatory ability of the NLTFe hydrogel. Furthermore, granulation tissue (G) formation was observed in all groups at day 3, while advanced matrix remodeling toward neodermis (ND) became evident at day 11 in treated groups, particularly in the NLTFe group. Notably, complete re‐epithelialization (NE) was only observed in the NLTFe group. These observations implied that the NIR‐assisted LTFe hydrogel was critical for promoting wound healing.

**FIGURE 8:**
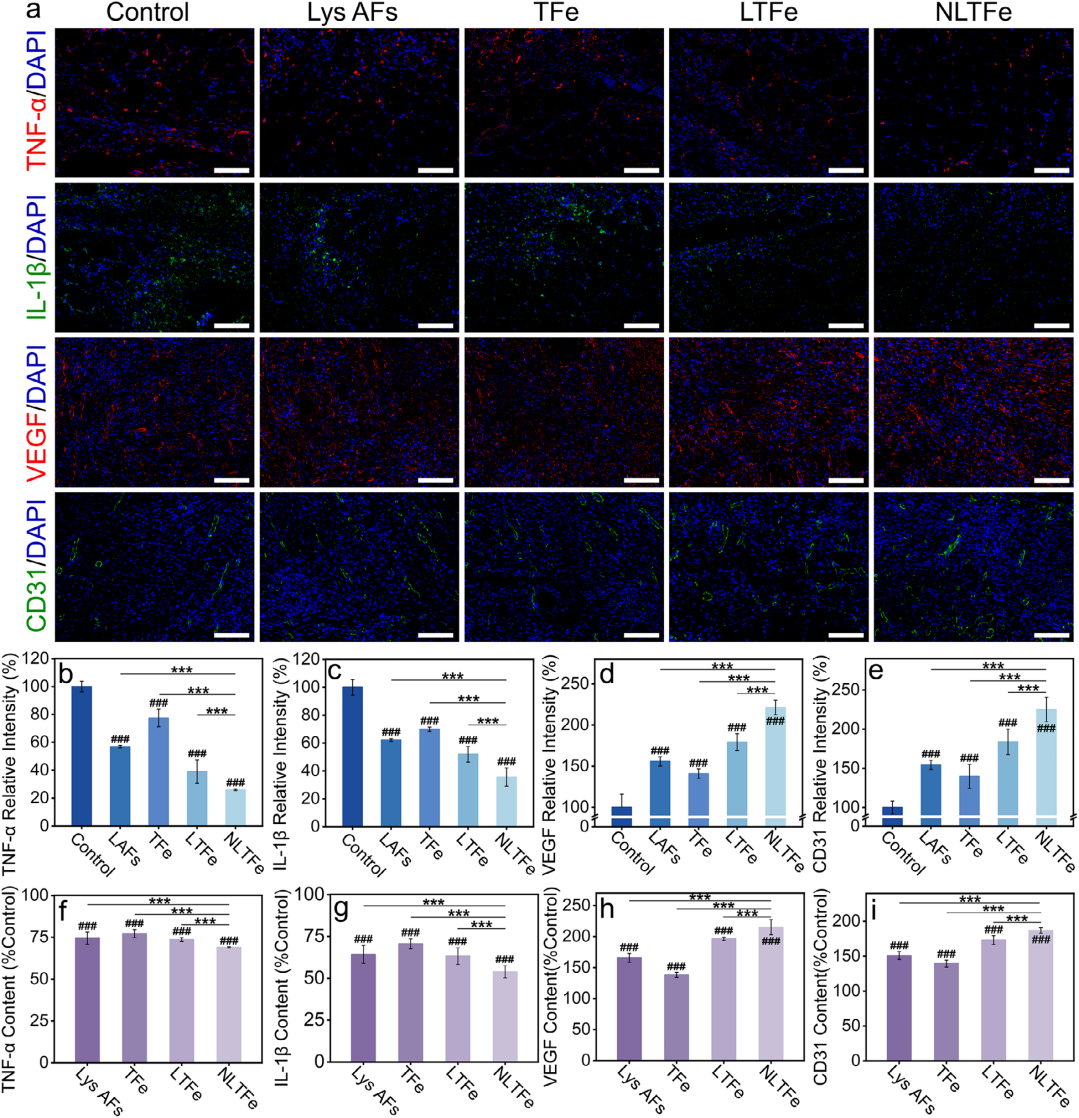
(a) TNF‐α (red), IL‐1β (green), VEGF (red), and CD31 (green), colocalized with DAPI stained nuclei (blue) immunofluorescent staining. (b) Corresponding quantification of the relative TNF‐α (b), IL‐1β (c), VEGF (d), and CD31 (e) expression. (c) ELISA assay of the relative TNF‐α (f), IL‐1β (g), VEGF (h), and CD31 (i) expression in different treatment groups: control, Lys AFs, TFe composite, LTFe‐ and NLTFe‐based hydrogels. Scale bars: 100 µm. *Note*: ^*^ *p* < 0.05, ^**^ *p* < 0.01, and ^***^ *p* < 0.001 vs. LTFe hydrogel; ^#^ *p* < 0.05, ^##^ *p* < 0.01, and ^###^ *p* < 0.001 vs. the control group, *n* ≥ 3.

To further demonstrate the anti‐inflammatory effects, immunofluorescent staining assays for two typical proinflammatory cytokines, TNF‐α and IL‐6, are further displayed in Figure 8a. As shown, high expressions of TNF‐α and IL‐6 were detected at day 3 in the control group, which suggested a severe inflammatory response. It is worth noting that we observed a significant decrement in the expression of TNF‐α and IL‐6 in Lys AFs, TFe, and LTFe groups, while negligible secretion was observed for NLTFe treatment. It is clearly evident from the quantitative results in Figure 8b,c that proinflammatory cytokines were significantly decreased by 25.9% (for TNF‐α) and 35.5% (for IL‐6) over the control group (*p* < 0.001). Similar changes were shown by the ELISA assay in Figure 8f,g. Of note, LTFe hydrogel also possessed intrinsic antioxidant properties conferred by the TFe network (Figure 3d). This antioxidant capacity was expected to suppress excessive inflammation and create a favorable microenvironment for tissue repair, thereby synergistically contributing to the wound healing process. These results provided strong supportive evidence that all treatments achieved significant anti‐inflammatory effects as compared to the control group, while the NIR‐assisted NLTFe group appeared to achieve the greatest such effects.

The wound healing process always involves an angiogenesis phase. Accelerated angiogenesis can further promote wound closure [40]. To assess angiogenesis capacity, overexpression of vascularization‐related genes (VEGF and CD31) was detected by immunofluorescent staining (Figure 8a). Representative staining images showed dramatically elevated VEGF‐ and CD31‐positive cells for all treatments. As anticipated, promoted angiogenesis proportions of NLTFe group were up to ∼2.21 times (VEGF) and ∼2.25 times (CD31) over the control, as determined by the corresponding quantitative data in Figure 8d,e. Additionally, ELISA assay showed that the NLTFe‐treated group exhibited significantly upregulated expression levels of VEGF and CD31 in wound tissues compared to the other groups (Figure 8h,i). These experimental results indicated that the photothermal‐activated NLTFe composite material fully leverages the synergistic effects of material properties and photothermal effects, efficiently promoting wound vascular formation by upregulating VEGF and CD31 expression, thereby accelerating the wound healing process.

## 4 Conclusion

This work offers a significant advance in the design of multifunctional, photothermal‐activated hydrogels for promoting wound healing. Our LTFe supramolecular hydrogel, generated through Fe^3+^‐mediated coordination between amyloid fibrils (Lys AFs) and tannic acid (TA) upon heating, represents a multifunctional biomaterial with a remarkable spectrum of properties. The system combines highly efficient and stable photothermal conversion capabilities, enabling robust performance under near‐infrared (NIR) irradiation, with potent antibacterial effects that arise both intrinsically from its chemical composition and synergistically through photothermal enhancement. Beyond its antimicrobial efficacy, the hydrogel demonstrates excellent biocompatibility, ensuring safe interaction with host tissues, while also exhibiting pronounced anti‐inflammatory activity that mitigates excessive immune responses and reduces tissue damage. In parallel, its pro‐angiogenic properties actively stimulate new blood vessel formation, a critical factor in tissue regeneration. Collectively, these complementary features work in concert to significantly accelerate the repair and regeneration of infected wounds in vivo, positioning the LTFe hydrogel as a powerful therapeutic platform for advanced wound management.

Looking forward, we envision that the same formulation can be adapted into various structural formats to broaden its clinical applicability. For example, sprayable or 3D‐printed configurations based on this hydrogel system may enable minimally invasive administration and patient‐specific wound dressing designs, as has recently been explored in related biomaterial research [41, 42]. Nevertheless, the long‐term storage stability of these formulations requires further evaluation before clinical translation.

## Author Contributions

**Di Wu**: investigation, conceptualization, methodology, validation, formal analysis, data curation, writing – original draft, writing – review and editing. **Jiangtao Zhou**: investigation (AFM measurement), conceptualization, supervision, writing – review and editing. **Yang Shen**: investigation (conducting in vitro experiments), validation, and formal analysis. **Qiyao Sun**: investigation (rheological measurement), data curation, and formal analysis. **Tong Li**: investigation (development and execution of in vivo mouse study), data curation, and formal analysis. **Xiaoyang Zou** and **Bin Liu**: investigation (molecular docking and molecular dynamics simulation), data curation, and formal analysis. **Hongshan Liang**: supervision, funding acquisition, project administration, and writing – review and editing. **Raffaele Mezzenga**: conceptualization, supervision, resources, and writing – review and editing.

## Conflicts of Interest

The authors declare no conflicts of interest.

## Supporting information




**Supporting File**: adhm71230‐sup‐0001‐SuppMat.docx.

## Data Availability

The data that support the findings of this study are available from the corresponding author upon reasonable request.
