# Supplementary material for: Photothermal‐Activated Antibacterial Amyloid‐Polyphenol‐Iron Hydrogels for Synergistic Wound Healing
Source: Adv Healthc Mater. 2026 May 10;15(26):e05910. doi: 10.1002/adhm.202505910 (PMC13356545; doi:10.1002/adhm.202505910)
Supplement: Supplementary file 1 — Supporting File: adhm71230‐sup‐0001‐SuppMat.docx. [file ADHM-15-0-s001.docx]

**Supplementary Information**

**Photothermal-Activated Antibacterial Amyloid-Polyphenol-Iron Hydrogels for Synergistic Wound Healing**

Di Wu^1,2,3^, Jiangtao Zhou^4^, Yang Shen^3^, Qiyao Sun^3^, Tong Li^1^, Xiaoyang Zou^1^, Bin Liu^3^, Hongshan Liang^1,*^, and Raffaele Mezzenga^3,5,*^

^1^College of Food Science and Technology, Huazhong Agricultural University, Wuhan, Hubei, 430070, China

^2^College of Food Science and Engineering, Qingdao Agricultural University, Qingdao 266109, China

^3^Department of Health Sciences and Technology, ETH Zurich, 8092 Zurich, Switzerland

^4^Department of Food Science & Technology, National University of Singapore, 2 Science Drive 2, 117542 Singapore

^5^Department of Materials, ETH Zurich, Wolfgang-Pauli-Strasse 10, 8093 Zurich, Switzerland

*Corresponding author: Hongshan Liang, Raffaele Mezzenga

E-mail address: [lianghongshan@mail.hzau.edu.cn](mailto:lianghongshan@mail.hzau.edu.cn); raffaele.mezzenga@hest.ethz.ch

**Table S1.** Nomenclature and composition of formulation under different TA/Fe concentrations.

| **Formulation** | **Lys** | **LTFe_1_** | **LTFe_2_** | **LTFe_3_** | **LTFe_4_** | **LTFe_5_** |
| --- | --- | --- | --- | --- | --- | --- |
| TA | - | 0.0175 mM | 0.175 mM | 0.35 mM | 0.7 mM | 1.4 mM |
| Fe^3+^ | - | 0.0175 mM | 0.175 mM | 0.35 mM | 0.7 mM | 1.4 mM |
| Lys AFs | 1% | 1% | 1% | 1% | 1% | 1% |

**Table S2.** Statistical analysis of the MM/GBSA results.

| **Energy**  **(kcal/mol)** | **Temperature** | |
| --- | --- | --- |
|  | **300 K** | **363 K** |
| Total | -38.18 | -27.93 |
| Coulomb | -105.2 | -90.86 |
| Covalent | 5.36 | -4.63 |
| Hbond | -1.97 | -0.76 |
| Lipo | -10.61 | -3.06 |
| Packing | -0.2 | -0.16 |
| SolvGB | 118.07 | 97.66 |
| vdW | -43.63 | -26.11 |


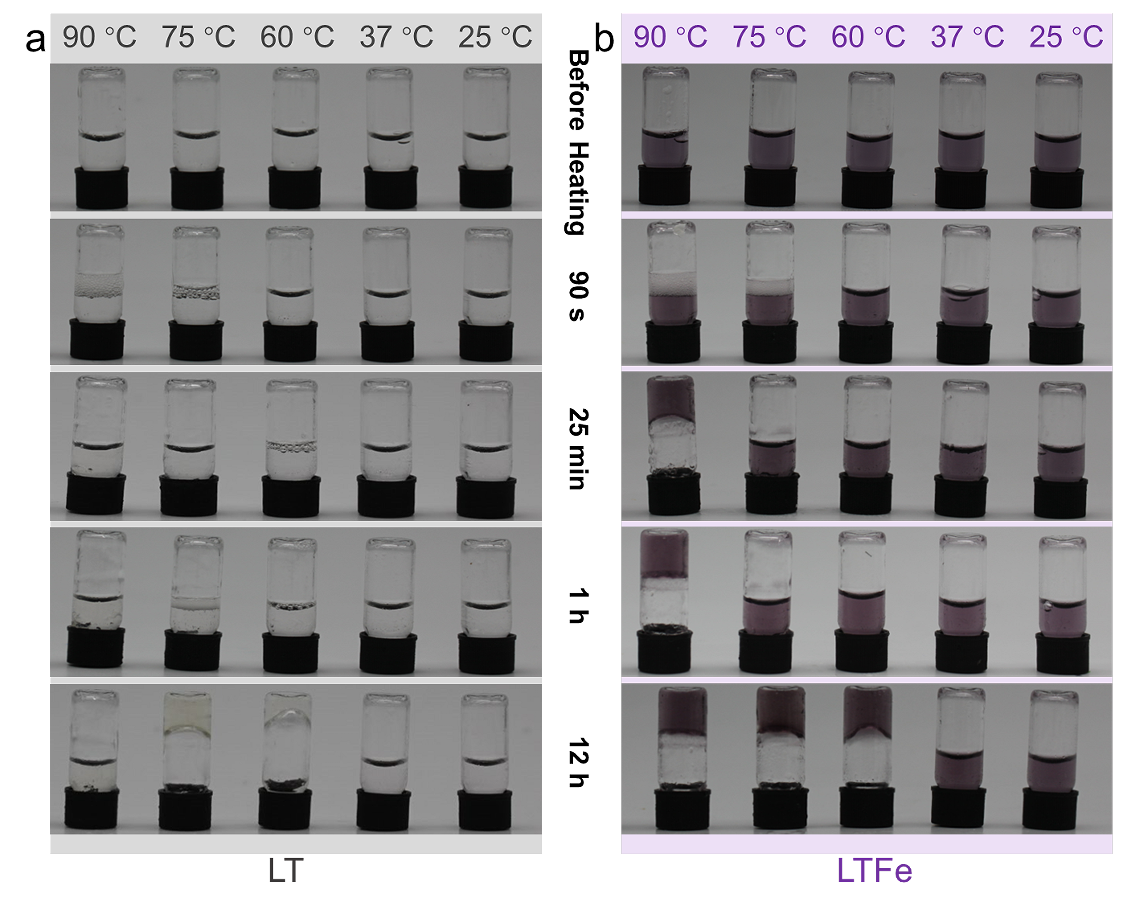


**Figure S1.** Upturn tests of **(a)** TFe composites and **(b)** LTFe composites under different temperatures with Lys AFs concentration of 1% and TFe molar mass of 175 μM.


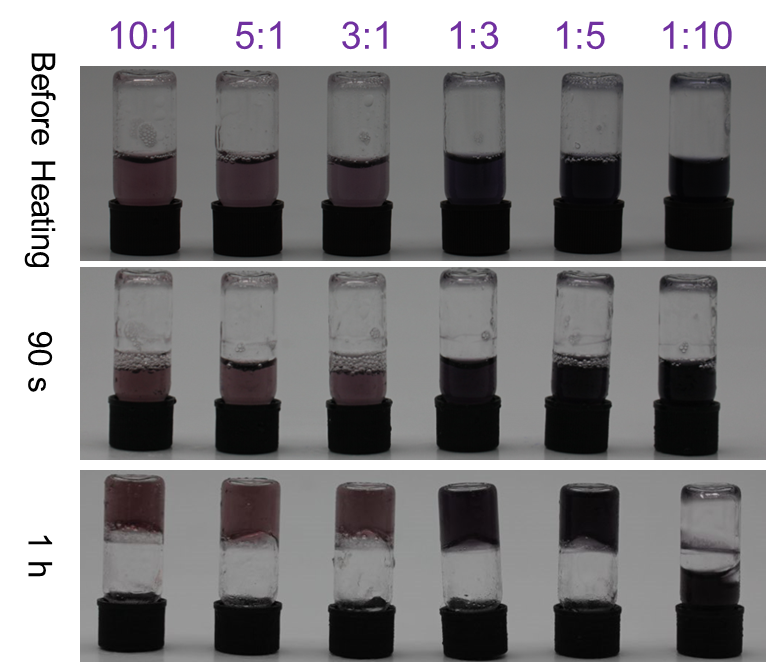


**Figure S2.** Upturn tests of LTFe composites under different ratios of TA to Fe^3+^ with Lys AFs concentration of 1%.


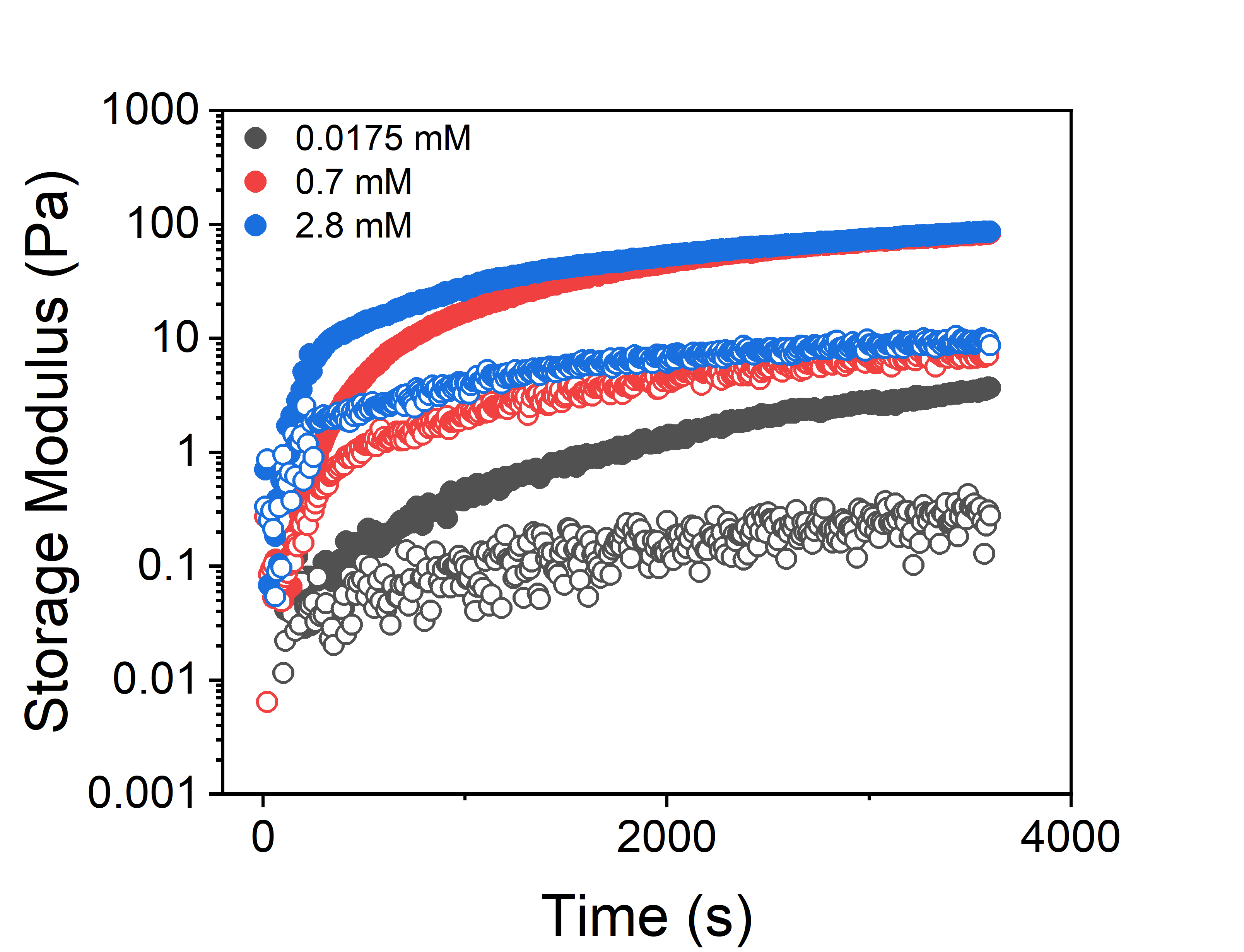


**Figure S3.** The storage modulus (G’, solid symbols) and loss modulus (G”, open symbols) as a function of time under different TFe molar mass.


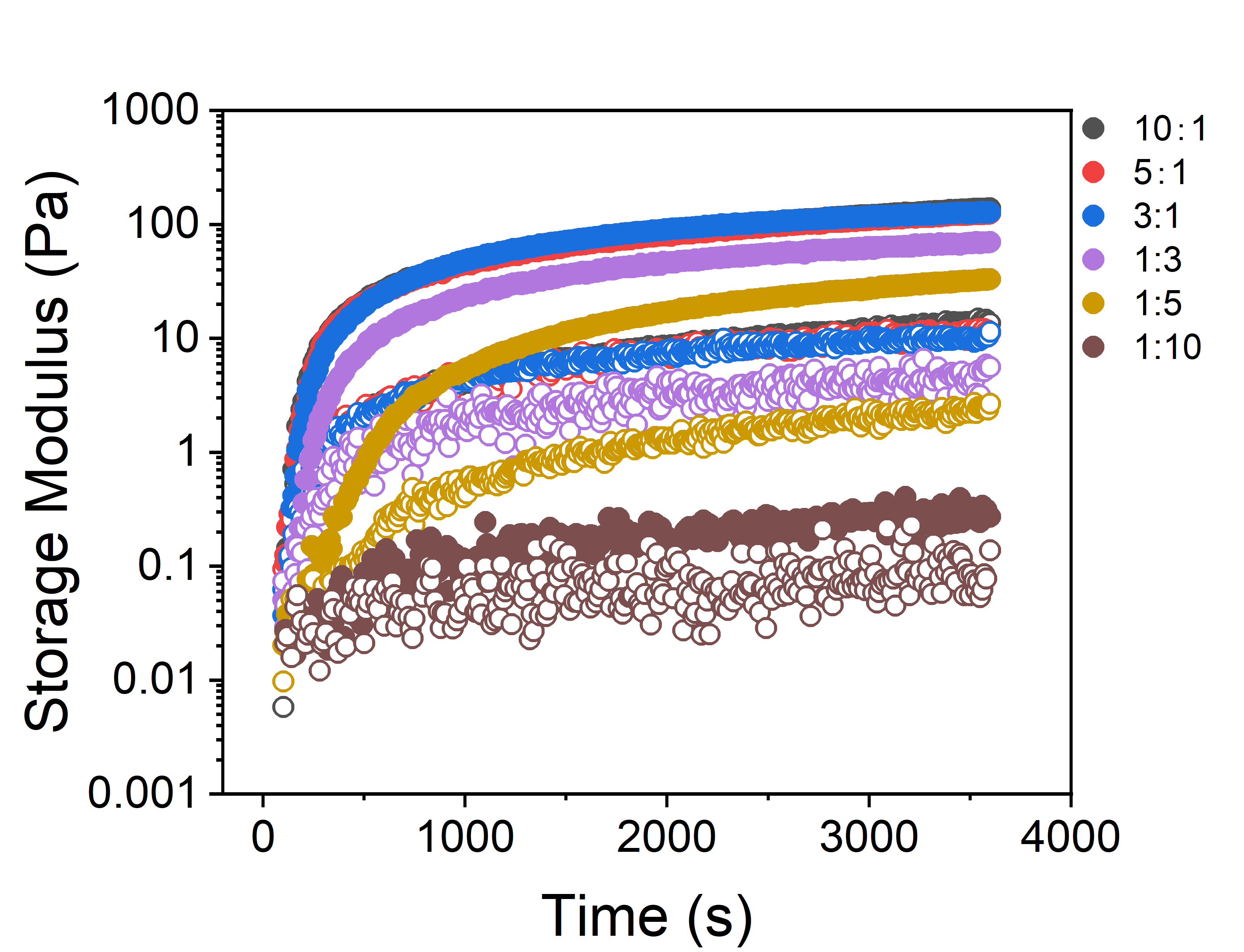


**Figure S4.** The storage modulus (G’, solid symbols) and loss modulus (G”, open symbols) as a function of time under different ratios of TA to Fe^3+^.


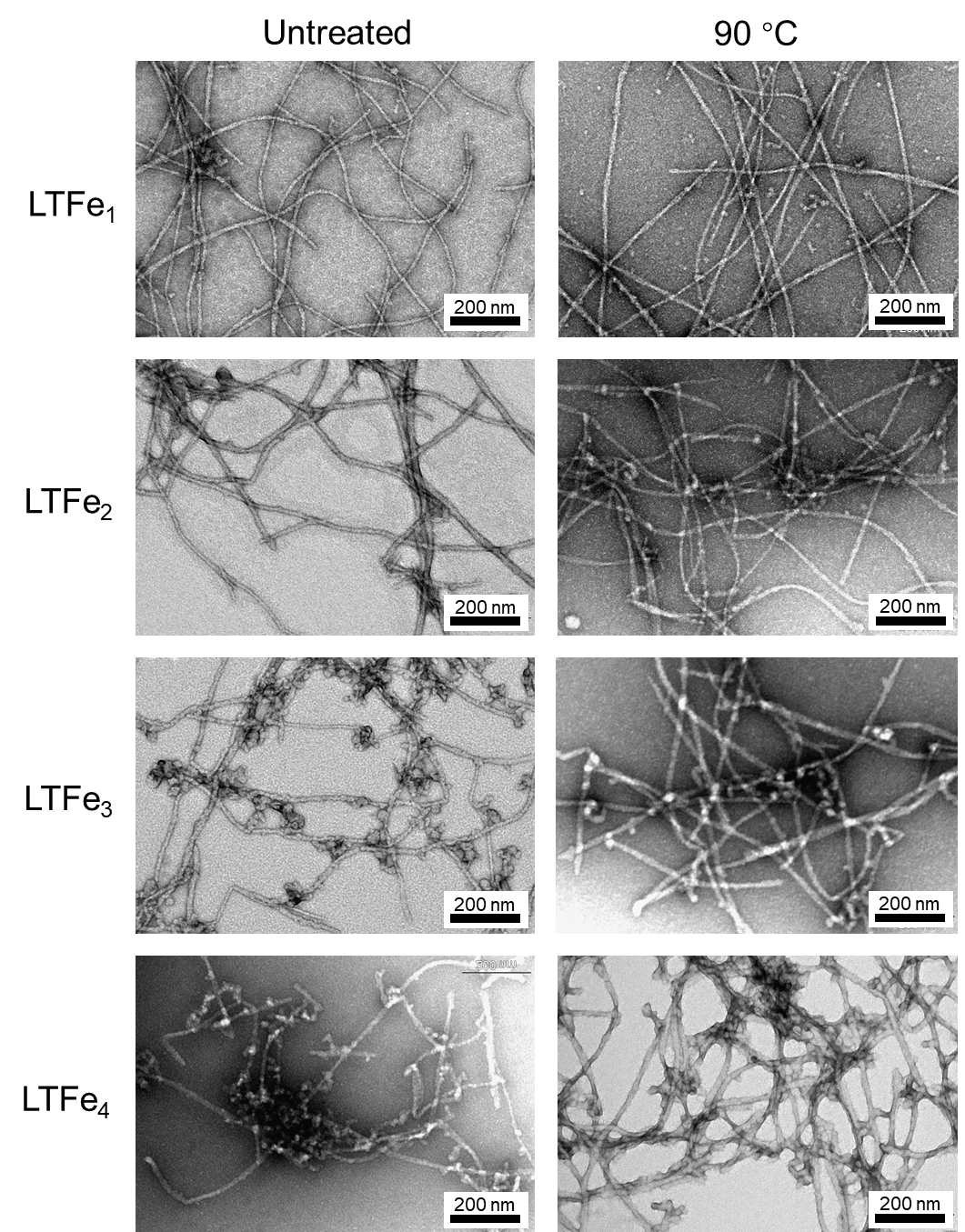


**Figure S5.** TEM images of LTFe composites before and after heating during 90 °C. Bar: 200 nm.


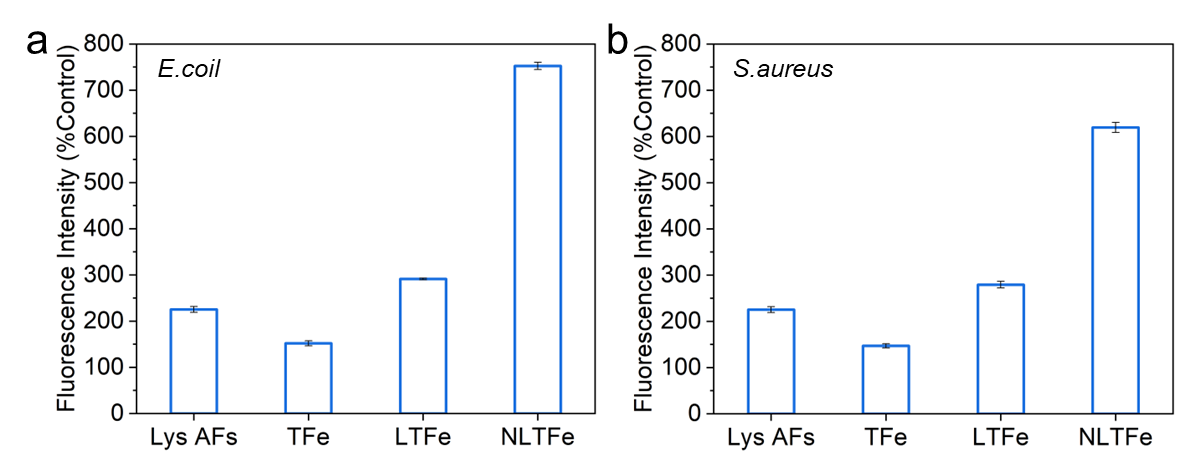


**Figure S6.** The intracellular ROS levels of *E. coli* **(a)** and *S.aureus* **(b)** treated by different formulations.


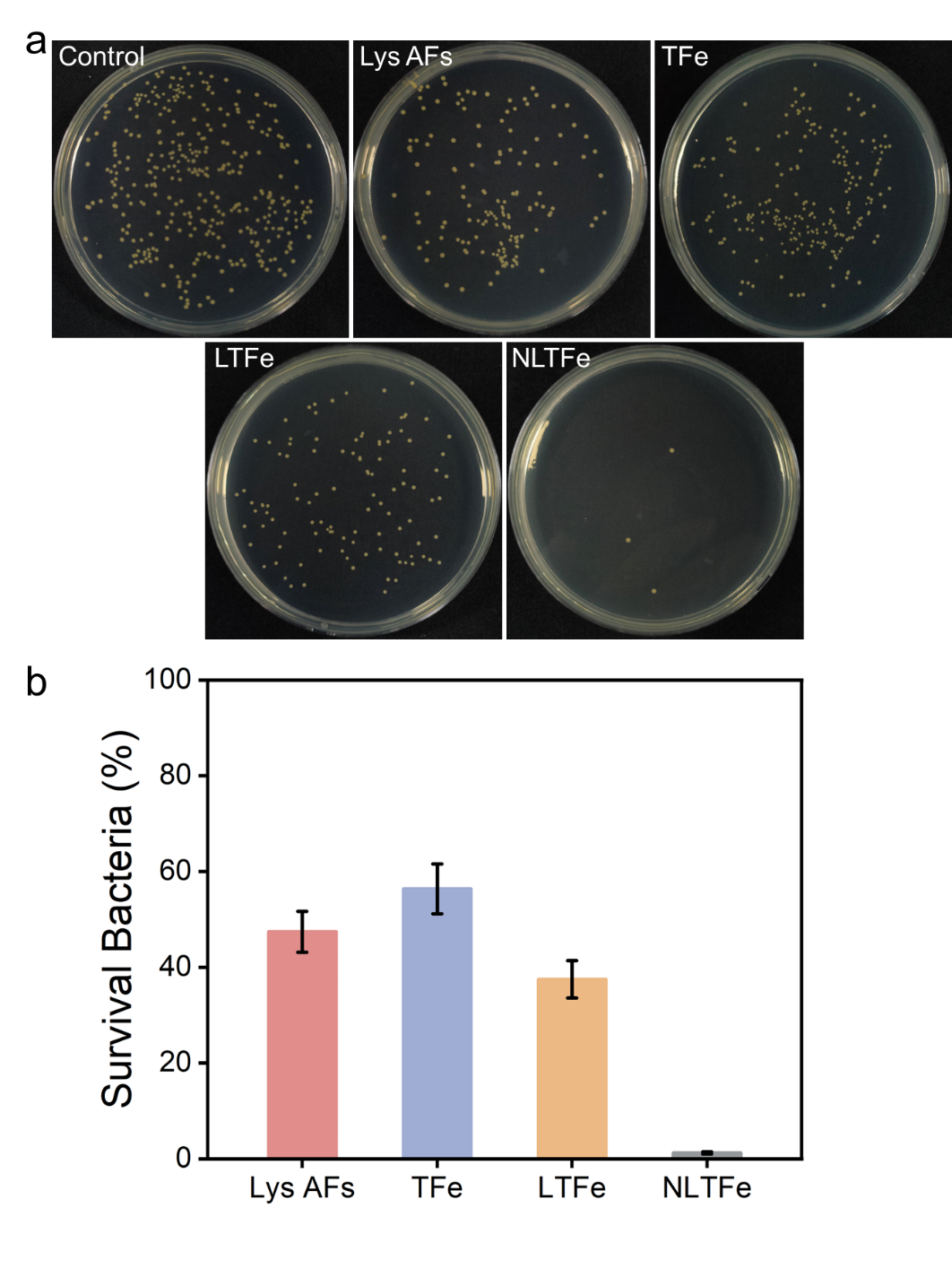


**Figure S7.** *In vivo* antibacterial examination. **(a)** Agar plates pictures and **(b)** corresponding statistical data of colonies of *S. aureus* treated with different formulations.
